# Prevalence and Antimicrobial Resistance of *Listeria monocytogenes* Isolated from Dairy Products in Romania

**DOI:** 10.3390/antibiotics14050482

**Published:** 2025-05-09

**Authors:** Filippos Georgios Nikolaou, Liora Mihaela Colobatiu, Laurentiu Mihai Ciupescu, Alexandra Tabaran, Ariana Raluca Hategan, Romolica Mihaiu, Radu Tanasuica, Magdalena Maria Poenaru, Marian Mihaiu

**Affiliations:** 1 Faculty of Veterinary Medicine, University of Agricultural Sciences and Veterinary Medicine Cluj-Napoca, 400372 Cluj-Napoca, Romania; nikolaou_filippos@yahoo.com (F.G.N.); alexandra.lapusan@usamvcluj.ro (A.T.); marian.mihaiu@usamvcluj.ro (M.M.); 2 Faculty of Pharmacy, Iuliu Hatieganu University of Medicine and Pharmacy, 400012 Cluj-Napoca, Romania; 3 Institute of Hygiene and Veterinary Public Health, 041293 Bucharest, Romania; ciupescu_laurentiu@yahoo.com; 4 National Institute for Research and Development of Isotopic and Molecular Technologies, 400293 Cluj-Napoca, Romania; ariana.hategan@itim-cj.ro; 5 Faculty of Economic Sciences and Business Administration, Babes-Bolyai University, 400591 Cluj-Napoca, Romania; romolica.mihaiu@econ.ubbcluj.ro; 6 Faculty of Veterinary Medicine, University of Agronomic Sciences and Veterinary Medicine Bucharest, 011464 Bucharest, Romania; radu.tanasuica@gmail.com; 7 Faculty of Horticulture, University of Craiova, 200585 Craiova, Romania; poenaru_magdalena@yahoo.com

**Keywords:** *Listeria monocytogenes*, dairy products, prevalence, antimicrobial resistance, serogroup distribution, Romania

## Abstract

**Background/Objectives:** *Listeria monocytogenes* is a significant foodborne pathogen associated with dairy products, which can pose serious public health risks, particularly for vulnerable populations. This study aimed to assess the prevalence, serotype distribution, and antimicrobial resistance profiles of *Listeria monocytogenes* isolated from dairy products collected in Romania over a three-year period (2021–2023). To the best of our knowledge, this is the first comprehensive study addressing these issues within the country. **Methods**: A total of 10,306 dairy samples, including milk, cheeses, ice cream, yogurt, and other dairy-based products, were collected and analyzed using standard microbiological methods. Molecular serotyping was performed to identify the most common serogroups. The antimicrobial susceptibility of the isolates was also conducted. **Results**: The overall prevalence of *Listeria monocytogenes* was 0.41% (43/10,306). The most frequently detected serogroup was IVb (74.41%), followed by IIa (23.25%) and IIb (2.32%). Ice cream was the most affected product, followed by fresh telemea made from cow milk. Antimicrobial susceptibility testing revealed higher resistance rates for oxacillin and trimethoprim-sulfamethoxazole (13.95% each), while all isolates were susceptible to ciprofloxacin, levofloxacin, and moxifloxacin. **Conclusions**: The findings emphasize the need for continuous monitoring of *Listeria monocytogenes* in dairy products, particularly ice cream and fresh cheeses, due to their high contamination rates. The study’s results are valuable for comparative analysis with findings from other countries, helping to establish a broader understanding of *Listeria monocytogenes* contamination trends and resistance profiles.

## 1. Introduction

*Listeria monocytogenes* (*L. monocytogenes*) is a ubiquitous bacterium that is widely distributed in the environment, a genetically heterogeneous species divided into 13 serotypes and four phylogenetic lineages, of which lineages I and II are the most frequently encountered [1,2].

*L. monocytogenes* is pathogenic to both humans and animals, being transmitted to the consumer mainly via contaminated ready-to-eat (RTE) foods [3]. There are multiple pathways through which *L. monocytogenes* can infiltrate the RTE food chain. Among these, soil and water are regarded as the primary reservoirs for the bacterium, facilitating its transmission to plant-based materials, animal feed, livestock, and, ultimately, the broader food production system [3]. This pathogen is highly resilient, capable of persisting in soil environments for extended periods—sometimes spanning several months—and even proliferating under conditions that support its growth [4]. It also has the ability to form biofilms, therefore increasing its resistance to disinfectants and complicating contamination control. The agricultural environment, particularly farms, represents a significant and continuous source of *L. monocytogenes* contamination, contributing to its introduction into raw food materials [5]. The raw materials originating from primary production play a crucial role in determining the presence of the pathogen in the final product, as they serve as potential initial sources of contamination during processing [2].

The processing of RTE foods involves various techniques, including heating, preservation, decontamination, and fermentation, many of which might help reduce *L. monocytogenes* contamination. However, the effectiveness of these methods depends on the process intensity, with mild treatments allowing survival and more rigorous ones eliminating the pathogen. A significant risk is represented by recontamination during post-processing and handling (increased handling also increases the probability of contamination) [2].

*L. monocytogenes* is responsible for severe foodborne infections in humans, invasive forms of infection being mostly manifested as septicemia, meningitis, or spontaneous abortion. It is also capable of causing invasive disease in a wide range of animal species [1,4,6].

In recent years, multiple food industries across Europe have faced significant disruptions due to outbreaks of foodborne illnesses associated with *L. monocytogenes* [1,6,7].

In 2019, listeriosis was the most severe zoonotic disease, recording the highest case fatality rate (13%) among all infections linked to outbreaks. In 2022, listeriosis was the fifth most reported zoonosis in the European Union (UE), with 2738 cases, reflecting a 15.9% rise in the notification rate compared to 2021 [6,8,9]. In 2023, in the EU, 27 Member States reported 2952 confirmed cases of invasive *L. monocytogenes* infections in humans, resulting in a notification rate of 0.66 cases per 100,000 people. This marked a 5.8% rise compared to the 2022 rate (0.63 cases per 100,000) and represented the highest number of cases and notification rate recorded since 2007. Notably, the overall trend for *L. monocytogenes* infections showed a statistically significant increase between 2019 and 2023. Among the reporting countries, Romania and Croatia registered the lowest notification rate (≤0.20 per 100,000 people) [6,10].

In general, the most sampled RTE food categories for the detection and/or enumeration of *L*. *monocytogenes* are RTE meat and meat products, as well as RTE milk and milk products. Based on the EU One Health 2023 Zoonoses Report, *L. monocytogenes* occurrences reported from official sampling were generally low, ranging from 0% to 2.8% at both the manufacturing and distribution stages. However, at the distribution level, an exception was noted for products of meat origin and fermented sausages, which showed a moderate positive proportion of 14.8%, followed by hard cheeses (1.8%), products of meat origin other than fermented sausages (1.4%), and fish (1.1%). At the manufacturing stage, the highest occurrences of *L. monocytogenes* were reported in fish and fishery products (1.8%), followed by soft and semi-soft cheeses (1.3%) [6,10].

*Listeria* species are generally susceptible to a wide range of antimicrobial agents.

Currently, the standard treatment for human listeriosis consists of a β-lactam antibiotic, such as ampicillin or penicillin, combined with an aminoglycoside like gentamicin. Alternative therapies may include vancomycin, erythromycin, and trimethoprim-sulfamethoxazole (especially recommended in the case of patients with β-lactam allergies or pregnant women) [8,11,12,13].

Antimicrobial-resistant isolates of *L. monocytogenes* have been detected in food, environmental sources, and human listeriosis cases. Recent studies have identified *L. monocytogenes* strains in dairy products exhibiting resistance to several antibiotics, including ampicillin, penicillin G, tetracycline, and chloramphenicol. The increasing resistance of *L. monocytogenes* to key therapeutic agents has become a growing concern, particularly given their central role in the treatment of invasive listeriosis [14].

Several mechanisms contribute to the antimicrobial resistance observed in *L. monocytogenes*, particularly strains isolated from food sources such as dairy products, including the acquisition of resistance genes through plasmids and transposons, the formation of persister cells and biofilms, or the activity of efflux pumps [15,16]. Antimicrobial resistance compromises the effectiveness of standard therapies for listeriosis, a disease associated with high morbidity and mortality, particularly among vulnerable populations. Furthermore, the pathogen’s ability to persist in food-processing environments through biofilm formation and resistance gene acquisition increases the risk of contamination of RTE products, such as dairy foods, leading to potential outbreaks that are more difficult to control and treat.

In Romania, there is a limited number of studies assessing the prevalence and antibiotic resistance of *Listeria monocytogenes* in RTE foods. Among these, a recent one investigated the prevalence, serotype distribution, virulence gene profiles, and antimicrobial resistance patterns of *L. monocytogenes* in 8151 samples from dairy, meat, and fish products, reporting an overall prevalence of 0.31%, most positive samples being detected in pork meat (42.3% of the positive samples). Only one *L. monocytogenes* isolate was recovered from dairy (raw milk cheese) [9].

The prevalence and resistance patterns of *Listeria* spp. may be shaped by antibiotic usage practices and regional differences in antimicrobial policies. Factors such as agricultural antibiotic use, food production hygiene standards, and environmental conditions contribute to variations in resistance profiles across different geographical regions. Comprehensive surveillance of *Listeria* spp. is essential for public health protection, as systematic monitoring enables the identification and characterization of clinically relevant serotypes such as 1/2a, 1/2b, and 4b, which are responsible for the majority of human listeriosis cases. It can also facilitate the early detection of resistant strains and help monitor emerging resistance trends [9,17,18].

Romania boasts a rich tradition of cheese and other dairy products that are integral to its culinary heritage. To the best of our knowledge, no comprehensive study has been conducted on dairy samples in Romania so far. Therefore, the current study aimed to evaluate the prevalence of *L. monocytogenes* in dairy samples collected in Romania over a period of three years (2021–2023), as well as to characterize the serotype distribution and antibiotic susceptibility profiles of the isolated strains using both classical and molecular methods.

## 2. Results

### 2.1. Prevalence of L. monocytogenes in Dairy Samples

**Figure 1:**
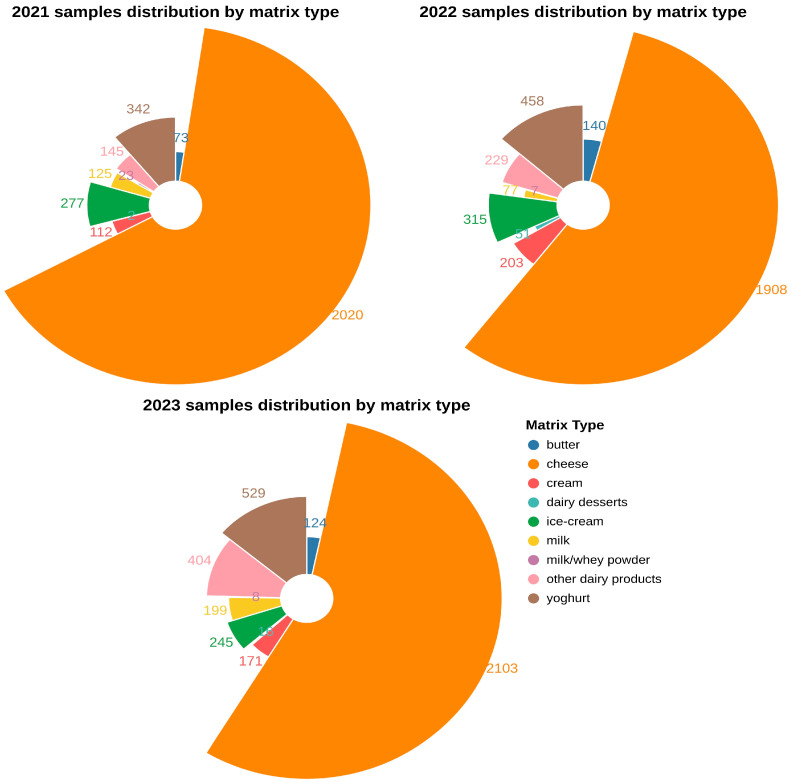
Samples distribution by matrix type throughout the study period (2021–2023).

A total of 10,306 dairy samples were collected throughout the study period (2021–2023), with cheese being consistently the most frequently collected sample type across all three years. A detailed representation of the samples’ distribution by matrix type is illustrated in Figure 1.

**Figure 2:**
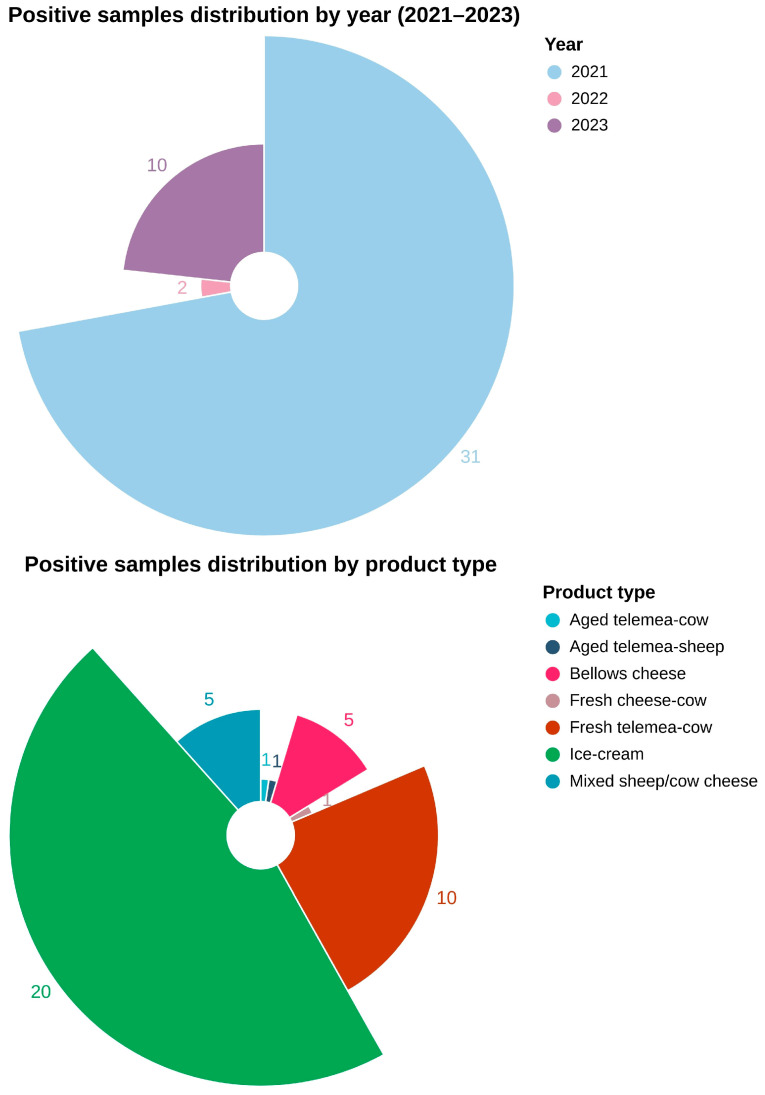
Distribution of positive samples by year and by type of dairy product.

Forty-three isolates of *L. monocytogenes* were recovered from the total number of samples included in the study (n = 10,306), resulting in an overall prevalence of 0.41% (43/10,306; 0.41%). Among these, during the year 2021, a total of thirty-one positive isolates were identified, which represents the highest count of positive samples recorded throughout the study period. A total of ten positive samples were detected in 2023, while in 2022, only two positive isolates were identified, marking the lowest incidence of positive samples across all three years (Figure 2).

Based on the distribution of positive samples according to the type of dairy product analyzed, ice cream accounted for the highest number of positive samples (20/43), followed by fresh telemea obtained from cow milk (10/43), aged telemea cow cheese (5/43), and bellows cheese (5/43) (Figure 2).

The highest number of positive samples originated from Dâmbovița County (22 samples), followed by Alba and Covasna Counties (10 samples each), while a single positive sample was identified in Galați County.

**Figure 3:**
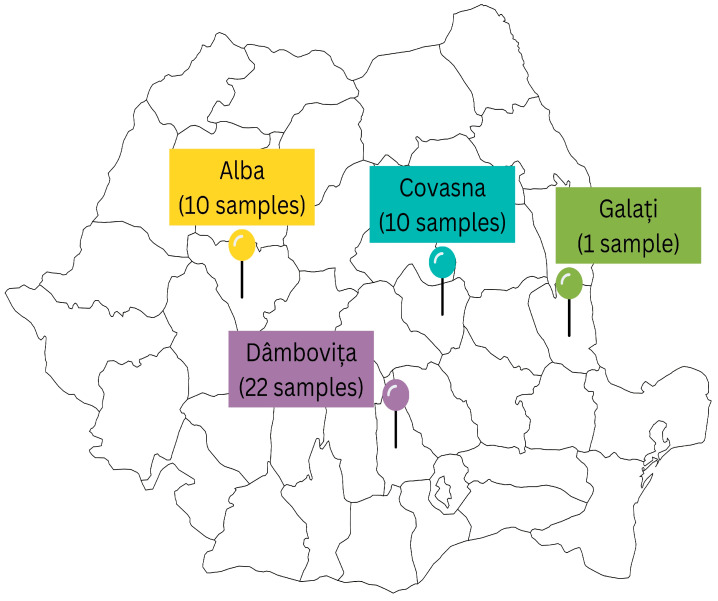
Geographical distribution of *L. monocytogenes*-positive dairy samples in Romania.

Figure 3 illustrates the regional origin of dairy samples that tested positive for *L. monocytogenes*. A total of 43 positive samples were distributed across four counties in Romania.

**Figure 4:**
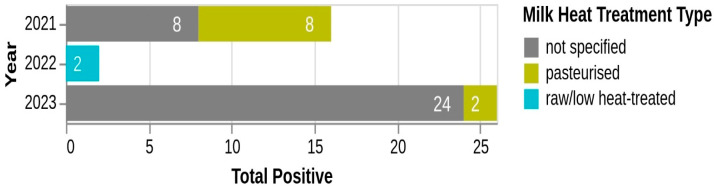
Distribution of *L. monocytogenes*-positive samples by milk heat treatment type.

The distribution of *L. monocytogenes* positive samples according to milk heat treatment type over the three-year period (2021–2023) is as follows: In 2021, positive samples were evenly distributed between milk and dairy products obtained from pasteurized milk (eight samples; 50%) and products with unspecified heat treatment (8 samples; 50%). In 2022, all positive samples (two samples) were associated with raw or low heat-treated milk. In 2023, most positive samples (24 samples; 92.3%) were from products with unspecified milk heat treatment, while a smaller proportion (two samples; 7.7%) were linked to pasteurized milk (Figure 4).

### 2.2. Molecular Serotyping of L. monocytogenes

**Figure 5:**
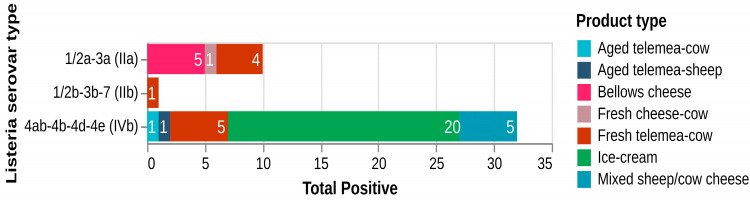
Distribution of L. monocytogenes serogroups among contaminated dairy matrices.

**Figure 6:**
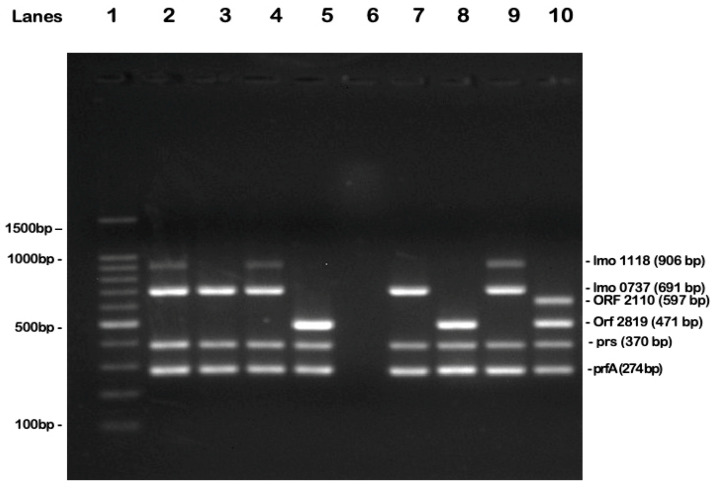
Multiplex PCR serotyping of *L. monocytogenes* strains. Lanes 1 to 10 represent the following: Lane 1—DNA ladder (molecular sizes in base pairs indicated on the right); Lane 2—sample positive for serogroup IIc; Lane 3—sample positive for serogroup IIa; Lane 4—sample positive for serogroup IIc; Lane 5—sample positive for serogroup IIb; Lane 6—negative control; Lane 7—positive control for serogroup IIa; Lane 8—positive control for serogroup IIb; Lane 9—positive control for serogroup IIc; Lane 10—positive control for serogroup IVb (obtained from the Listeria Culture Collection of the National Reference Center for Listeria, Institut Pasteur, Paris); The molecular sizes of the amplified genes are indicated (in base pairs) on the left side of the gel image.

Three major serogroups were associated with the isolated strains of *L. monocytogenes*, namely IIa, IIb, and IVb, each exhibiting varying levels of contamination across different dairy matrices (Figure 5 and Figure 6).

Serogroup IIa (1/2a-3a) was associated with a total of 10 positive samples, representing 23.25% of all positive samples identified (10/43; 23.25%). This serogroup was mainly associated with soft and fresh dairy products. It was most frequently detected in bellows cheese (five positive samples), followed by fresh telemea from cow milk (four positive samples), and in one case, fresh cheese obtained from cow milk (one sample). These findings indicate that serogroup IIa was present in both traditional and industrial cheese types, with a relatively balanced distribution among the sampled cheese categories.

Serogroup IIb (1/2b-3b-7) was the least frequently detected, with only one positive sample identified in fresh telemea obtained from cow milk (1/43; 2.32%). No other product types included in the study yielded IIb-positive results, suggesting a limited occurrence within the sampled dairy products.

In contrast, serogroup IVb (4ab-4b-4d-4e) exhibited the highest prevalence, being detected in 32 of the 43 positive samples distributed across various dairy matrices (32/43; 74.41%). Notably, ice cream was the most affected product, accounting for 20 out of the 32 positive samples linked to this serogroup. The remaining IVb-positive samples were distributed among fresh cheese (five samples), fresh telemea (four samples), and bellows cheese (three samples). This serogroup was therefore detected across all sampled product types, but with a disproportionately high occurrence in ice cream.

Overall, the distribution of serogroups varied considerably by product type. While serogroup IIa was mostly associated with cheese samples, particularly bellows cheese and telemea, serogroup IVb was dominant in both frequency and product diversity, particularly in frozen dairy products. Serogroup IIb was only sporadically detected.

### 2.3. Antimicrobial Susceptibility

The antimicrobial susceptibility testing of *L. monocytogenes* isolates revealed varying levels of resistance to different antimicrobial agents, as summarized in Table 1. The highest resistance rates were observed for oxacillin and trimethoprim-sulfamethoxazole, with 6 out of 43 (13.95%) isolates exhibiting resistance to each. Tetracycline and ampicillin also demonstrated relatively high resistance rates at 11.62% each (five isolates), while penicillin G exhibited a resistance rate of 9.30% (four isolates). These findings are of note as ampicillin and penicillin G are first-line agents in the treatment of human listeriosis, often administered in combination with gentamicin for its synergistic bactericidal effect in particular cases. Lower resistance rates were detected for methicillin, clindamycin, gentamicin, chloramphenicol, and rifampicin, each accounting for 2.32% of resistant isolates (one isolate). No resistance was detected against ciprofloxacin, levofloxacin, or moxifloxacin. Additionally, intermediate resistance was noted for oxacillin (4.65%), ampicillin (2.32%), penicillin G (2.32%), and trimethoprim-sulfamethoxazole (2.32%). 

Overall, the data reveal that while the majority of *L. monocytogenes* isolates remained susceptible to key antimicrobials, a subset demonstrated resistance—most notably to drugs that are either central to listeriosis treatment or serve as important alternatives in cases of beta-lactam allergy. 

## 3. Discussion

The dairy industry is one of the largest and most dynamic sectors within the global food industry, producing a diverse array of perishable and semi-perishable products that are in high demand worldwide, including milk, but also a wide variety of processed dairy products such as cheese, butter, yogurt, cream, ice cream or powdered milk, each with unique processing requirements and safety considerations [14,19].

Due to their rich nutritional composition, including high levels of proteins, fats, vitamins, and minerals, dairy products provide an ideal environment for the growth and proliferation of a wide variety of microorganisms. These favorable conditions support not only beneficial microbes but also undesirable organisms, including spoilage bacteria, molds, yeasts, and pathogenic bacteria. Contamination can occur at any point along the dairy production chain, from the initial milking process through processing, packaging, storage, distribution, and even during handling by consumers [20,21].

The presence of spoilage-inducing and pathogenic microorganisms in dairy products is particularly problematic because it can compromise both the safety and shelf life of these products [14]. Notably, the contamination with *L. monocytogenes*, the causative agent of listeriosis, is of particular concern, as it is a highly adaptable and resilient pathogen, capable of surviving and growing under various environmental stressors commonly encountered in the food-production industry. It can proliferate at low temperatures (as low as −0.4 °C), endure repeated freezing and thawing cycles, and persist under adverse conditions, including a broad pH range (4.4–9.6), low water activity (<0.90), high osmotic pressure, UV light exposure, and the presence of biocides and heavy metals [22,23,24,25].

Even though *L. monocytogenes* is widely distributed in the environment and frequently isolated from a variety of foods, listeriosis is a less common foodborne disease compared to other foodborne illnesses such as salmonellosis. However, listeriosis is associated with elevated rates of hospitalization (more than 97%), as well as with a high fatality rate (up to 30%), even with appropriate antimicrobial treatment [25,26].

Studies investigating the prevalence of *Listeria* spp., including *L. monocytogenes*, have been conducted in many countries, across a wide range of food categories, including meat, seafood, milk, dairy products, eggs, and particularly RTE foods, which are considered high-risk due to their lack of further cooking or processing before consumption. Among RTE foods, dairy products such as soft cheeses (e.g., telemea, Brie, Camembert), ice cream, butter, and yogurt have received significant attention, being frequently implicated as potential vehicles of transmission [27]. To the best of our knowledge, no comprehensive study has been conducted in Romania to date regarding the prevalence of *L. monocytogenes* across a wide variety of dairy products. The current study involved the analysis of a significant number of dairy samples (10,306), including milk, milk-based drinks, cheese, cream, butter, yogurt, ice cream, dairy desserts, milk and whey powder, and other dairy products collected from across the country, over a period of three years (2021–2023), including both raw and processed dairy products. Forty-three isolates of *L. monocytogenes* were recovered from the total number of samples included in the study, resulting in an overall prevalence of 0.41% (43/10,306; 0.41%). The above-mentioned prevalence rate is lower compared to global estimates reported in a recent meta-analysis that incorporated findings from a total of 173 studies and found a pooled prevalence of *L. monocytogenes* in dairy products at 4.60% (95% CI: 1.72–8.60%) and even higher contamination in processing environments (8.69%, 95% CI: 5.30–12.78%) [27].

However, the prevalence rate reported in our study is slightly higher than the one reported by Duma et al. (2024) (0.31%), who investigated the prevalence of *L. monocytogenes* in RTE food products, including dairy products in Romania [9].

Our findings are partially consistent with the 2013 report by the European Food Safety Authority (EFSA), which analyzed data from 2010 to 2011 across the European Union. In this report, the prevalence of *L. monocytogenes* in soft and semi-soft cheeses at the end of their shelf life was reported to be 0.47% [28], a value comparable to our observed prevalence in similar product categories such as telemea and bellows cheese. While our overall prevalence across all dairy matrices was slightly lower (0.41%), the higher contamination rate observed specifically in soft cheeses in our study supports EFSA’s identification of these products as high-risk matrices. These similarities further emphasize the need for rigorous microbiological monitoring, especially in traditional and RTE cheese varieties, which are more susceptible to *L. monocytogenes* contamination due to their physicochemical properties and lack of heat treatment prior to consumption [28]. 

The analysis of the distribution of positive samples across different types of dairy products performed in our study revealed that ice cream exhibited the highest number of positive samples (20/43), followed by fresh telemea produced from cow milk (10/43) and aged telemea cow cheese and bellows cheese (“brânză de burduf” in Romanian-a traditional Romanian cheese made primarily from sheep’s milk) (5/43).

This distribution is notable, especially since *L. monocytogenes* is capable of surviving freezing conditions for extended periods. In line with this, Chen et al. [29] reported a major outbreak of listeriosis in the United States linked to contaminated ice cream, where over 99% of tested samples from a production line were positive. Although some studies (e.g., Ewida et al. found no contamination in ice cream, the survival capacity of *L. monocytogenes* under freezing conditions for up to 36 months reinforces the risk, particularly for vulnerable populations such as pregnant women, the elderly, and immunocompromised individuals [30].

Our data also support the notion that soft cheeses, including traditional Romanian varieties like telemea and bellows cheese, present a favorable environment for *L. monocytogenes* growth due to their high moisture content and neutral pH. These intrinsic properties, combined with the fact that such products are often consumed without further heat treatment, increase the potential for foodborne transmission [14,26,31].

The above-mentioned data highlights that even products unable to support bacterial growth, such as ice cream, can pose serious health risks if persistently contaminated at low levels, especially when consumed by highly susceptible populations [14,32].

When it comes to the importance of pasteurization in ensuring the safety of milk and its derivatives, the distribution of *L. monocytogenes*-positive samples according to milk heat treatment type over the three-year period (2021–2023) revealed a notable discrepancy in contamination rates. In 2021, positive samples were evenly distributed between pasteurized milk or pasteurized milk-derived products (50%) and products with unspecified heat treatment (50%), indicating possible cross-contamination or inadequate pasteurization processes. In contrast, in 2022, all positive samples were associated with raw or low heat-treated milk, highlighting the increased risk linked to insufficient heat treatment. Notably, in 2023, the vast majority of positive samples (92.3%) were from products with unspecified heat treatment, while only a small proportion (7.7%) were linked to pasteurized milk. This raises concerns about labeling accuracy, cross-contamination during post-processing, or inadequate sanitation practices in some facilities. The lower prevalence of positive samples from pasteurized milk or pasteurized milk-derived products, especially in 2023, suggests effective control measures when proper heat treatment is applied, but the high proportion of samples with unspecified treatment raises concerns about inadequate documentation of processing methods and potential contamination risks.

Also, it has been reported in different studies that the risk of *L. monocytogenes* contamination may persist even after pasteurization, due to improper temperatures, equipment malfunction during the process, or contamination occurring during subsequent production stages. Consequently, pasteurized dairy products may still harbor this microorganism. Research also suggests that *L. monocytogenes* enclosed within leukocytes in milk can survive when pasteurization temperatures are insufficient [33].

In light of the low overall prevalence observed in our study compared to international data, our findings may suggest relatively effective control measures within Romania’s dairy sector. However, the concentration of positive samples in specific product types—particularly those often consumed as RTE foods—warrants continued surveillance and stricter traceability protocols. Furthermore, the presence of *L. monocytogenes* in products with unclear thermal histories indicates the need for more rigorous documentation and regulatory oversight.

The identification of *L. monocytogenes* serogroups in the present study revealed the presence of three major serogroups: IIa, IIb, and IVb, each displaying distinct contamination patterns across various dairy matrices. These serogroups correspond to distinct evolutionary lineages and differ in their virulence potential, prevalence in food versus clinical cases, as well as their capacity to persist in the food chain. The most prevalent serogroup identified was IVb (4ab, 4b, 4d, 4e)**,** which accounted for 32 out of the 43 positive samples (74.41%)**,** predominantly isolated from ice cream (20 positive samples)**.** This finding is significant given that serotype 4b has been frequently associated with the majority of severe listeriosis outbreaks, including large-scale global incidents. According to Chen et al. (2020), although serogroup II.2 (1/2b-3b-7) was more common in food in China, serogroup II.1 (equivalent to IVb) remained the most closely associated with virulence strains, often harboring Listeria Pathogenicity Island 3 (LIPI-3) and LIPI-4, which are linked to enhanced neuroinvasion and placental tropism [30,34]. The dominance of this serogroup, particularly in ice cream, may suggest that products with extended storage under refrigeration, combined with inadequate handling or post-processing contamination, may contribute to the high occurrence of this lineage, underscoring the ability of the pathogen to survive extreme storage conditions.

Serogroup IIa (1/2a, 3a) was the second most prevalent, comprising 10 positive samples (23.25%), predominantly detected in soft and fresh cheeses such as bellows cheese (5 positive samples)**,** fresh cheese from cow milk (1 positive sample), and fresh telemea derived from cow milk (4 positive samples). This serogroup corresponds to Lineage II, which is commonly derived from environmental, agricultural, and food sources and frequently associated with non-clinical isolates. However, despite being primarily associated with environmental sources, several studies have demonstrated that serotype 1/2a is increasingly involved in sporadic and invasive human listeriosis, particularly in immunocompromised individuals [14,34,35]. Moreover, Lineage II strains have a strong ability to form biofilms and persist in food production environments, contributing to their recurrence in RTE foods, despite hygienic controls.

The least frequently detected serogroup was IIb (1/2b, 3b, 7), with only one positive sample (2.32%) isolated from fresh telemea derived from cow milk but is of notable importance due to its inclusion in Lineage I and association with virulent sequence types (e.g., ST87, ST1) [35,36]. It has also been reported that serogroup II.2 isolates frequently harbored both LIPI-1 and LIPI-3, indicating a strong potential for virulence even at low prevalence [30].

Together, our findings emphasize the dominance of the highly virulent Lineage I (particularly serogroup IVb) in dairy products, with significant implications for public health. These strains are capable of causing invasive disease and may be more difficult to eradicate from processing environments due to their resistance traits and survival mechanisms, including biofilm formation and stress adaptation systems.

Furthermore, the notable presence of Lineage II (serogroup IIa) in various fresh and soft cheeses highlights the need for stringent monitoring and control measures during production and handling, especially for traditionally made products that may not undergo extensive heat treatment. Public health interventions must also consider the serotype-specific risks to better anticipate and prevent listeriosis outbreaks.

The antimicrobial susceptibility testing of the *L. monocytogenes* isolates revealed varying levels of resistance against several antibiotics commonly used to treat listeriosis. The highest resistance rates were observed for oxacillin and trimethoprim-sulfamethoxazole, with 13.95% of isolates exhibiting resistance to each. Additionally, relatively high resistance rates were detected for tetracycline and ampicillin, each accounting for 11.62% of isolates, while penicillin G exhibited a lower resistance rate of 9.30%. These findings are clinically significant considering that ampicillin and penicillin G remain the cornerstone of treatment for invasive listeriosis. Resistance to these first-line agents may limit therapeutic options, particularly in severe cases.

Trimethoprim-sulfamethoxazole, often used as an alternative in patients allergic to β-lactams, also showed concerning resistance levels. Similar trends were reported by Duma et al. (2024), who found that resistance to trimethoprim-sulfamethoxazole and oxacillin reached 26.92% and 23.07%, respectively, in *L. monocytogenes* strains isolated from RTE food products in Romania [9].

Encouragingly, no resistance was detected against ciprofloxacin, levofloxacin, or moxifloxacin, which are generally effective against *L. monocytogenes*, consistent with prior findings and confirming the potential of these agents as second-line therapies in resistant cases. However, caution is warranted, as there is limited data on their efficacy in systemic listeriosis.

At the molecular level, tetracycline resistance is often associated with the detection of *tetC*, *tetM* and *tetK* genes, while the *ampC* gene is considered responsible for β-lactamase production and resistance to ampicillin. The gene *dfrD* confers resistance to trimethoprim.

The emergence of resistant and multidrug-resistant *L. monocytogenes* in the food chain, particularly in RTE dairy products, represents a significant public health threat. Contaminated foods may serve as reservoirs for strains that are not only virulent, but also less responsive to standard treatments, increasing the risk of therapeutic failure and complications in clinical cases Thus, continuous antimicrobial resistance monitoring, coupled with strict food safety controls and prudent antibiotic use in agriculture, is essential to safeguard the effectiveness of existing therapies and prevent the dissemination of resistant strains into the human population.

## 4. Materials and Methods

### 4.1. Sample Collection

This study analyzed a total of 10,306 dairy product samples—including milk, milk-based beverages, cheese, cream, butter, yogurt, ice cream, dairy desserts, milk and whey powders, and other dairy items—submitted to the Sanitary Veterinary and Food Safety Laboratories across Romania.

The primary objective was to assess compliance with microbiological safety standards, with a specific focus on the detection and confirmation of the absence of *Listeria* spp. in dairy products. 

The study was conducted over a three-year period (2021–2023), during which the number of samples tested per year was as follows: 3119 in 2021; 3388 in 2022; and 3799 in 2023.

All samples were delivered directly to the laboratories by producers, maintained in their original commercial packaging, and transported under refrigerated conditions (0–4 °C) to preserve microbiological integrity.

Samples were gathered from all administrative counties of Romania (during all seasons), providing comprehensive national representation in the assessment of *L. monocytogenes* contamination.

The heat treatment types included “not specified”, “pasteurized”, and “raw/low heat-treated milk”. Among these samples, 6545 were dairy samples derived from pasteurized milk, while for 3305 samples, the type or procedure of milk heat treatment was not specified. A total of 456 samples were represented by dairy samples derived from raw/low heat-treated milk.

### 4.2. Bacterial Isolation

The isolation of the *L. monocytogenes* strains was performed according to the horizontal detection and counting method, following the steps previously described by Duma et al. [9]. Briefly, a total of 25 g from each sample was inoculated into 225 mL of selective supplement half Fraser broth (Sharlau, Sentmenat, Spain) and incubated at 30 ± 1 °C for 25 ± 1 h. Following this, a second enrichment step was performed by transferring 0.1 mL of the broth culture into 10 mL of full-strength Fraser broth (UVM II Selective Supplement, Scharlau, Spain) and incubating it at 37 °C for 24 ± 2 h. After enrichment, a loopful from both half-strength and full-strength Fraser broths was streaked onto chromogenic ALOA agar (Scharlau, Spain) and Oxford agar (Merck, Darmstadt, Germany), followed by incubation under aerobic conditions at 37 °C for 24–48 h. Colonies displaying characteristic morphology on ALOA and Oxford agar were subsequently subcultured onto tryptic soy agar supplemented with 0.6% yeast extract (TSA-YE) (BioLife, Monza, Italy) and incubated at 37 °C for 24 h. For further identification, isolates were subjected to Gram staining, hemolysis testing on blood agar (Columbia Blood Agar Base, Oxoid, Basingstoke, UK, supplemented with defibrinated horse blood), carbohydrate utilization tests (Carbohydrates Utilisation Broth Base ISO, Condalab, Madrid, Spain), and CAMP tests using reference strains (*Staphylococcus aureus* ATCC 6538P, *Rhodococcus equi* ATCC 6939, *Listeria monocytogenes* ATCC 13932, *Listeria ivanovii* ATCC 19119, and *Listeria innocua* ATCC 33090). Final confirmation of *L. monocytogenes* strains was performed using biochemical testing with the help of the VITEK 2 GP Immunodiagnostic Assay System (Biomerieux, Craponne, France), following the manufacturer’s guidelines [9].

### 4.3. Antimicrobial Susceptibility Testing

Antimicrobial susceptibility was assessed based on the disk diffusion method, following the standard protocol recommended by the European Committee on Antimicrobial Susceptibility Testing (EUCAST), as previously described [9]. Following isolation, the frozen *L. monocytogenes* strains were thawed and plated onto Brain Heart Infusion (BHI) agar (Merck, Germany), then incubated at 37 °C for 24 h. Subsequently, 5–6 colonies from the overnight cultures were suspended in 1 mL of 0.9% NaCl solution, with the turbidity adjusted to a 0.5 McFarland standard. The prepared suspension was then inoculated onto Mueller–Hinton agar (Merck, Germany). Antibiotic disks were placed on the agar surface at 3 cm intervals, each containing a specific concentration of antimicrobials: β-lactams (ampicillin-10 μg, cephalothin-30 μg, penicillin G-10 μg, oxacillin-1 μg, and meticillin-5 μg), fluoroquinolones (ciprofloxacin-5 μg, levofloxacin-5 μg, moxifloxacin-5 μg), macrolides and lincosamides (clindamycin-2 μg), tetracyclines (tetracycline-30 μg), aminoglycosides (gentamicin-10 μg) and others (chloramphenicol-30 μg, rifampicin-30 μg, and trimethoprim-sulfamethoxazole-1.5/23.5 μg). The plates were subsequently incubated at 37 °C for 24 h, after which the diameter of the inhibition zones was measured to the nearest millimeter. Data interpretation followed EUCAST criteria [37]. In cases where EUCAST did not provide resistance breakpoints for *Listeria*, CLSI standards for *Staphylococcus aureus* and *Enterococcus* spp. were used as reference points [38].

### 4.4. Bacterial DNA Extraction

The bacterial DNA was extracted using the InstaGene Matrix kit (Bio-Rad, 732-6030, Sydney, Australia) following the manufacturer’s instructions with slight modifications.

Briefly, two to three bacterial colonies were resuspended in 200 μL of a 6% (*w*/*v*) Chelex^®^ resin solution provided with the Bio-Rad kit.

In the standard Bio-Rad protocol, a single colony is first suspended in 1 mL of distilled water, centrifuged to remove the supernatant, and then resuspended in the Chelex solution. In this study, that step was bypassed, and the colonies were directly suspended in the Chelex solution.

The samples were incubated at 56 °C for 30 min on a thermomixer set at 1400 rpm. The tubes were then heated at 98 °C for 15 min, whereas the Bio-Rad protocol recommends heating at 100 °C for only 8 min. Finally, the samples were centrifuged at 12,000 rpm for 5 min, compared to the Bio-Rad recommendation of centrifugation at 10,000–12,000 rpm for 2–3 min.

The resulting supernatant was stored at −18 °C until further use.

### 4.5. Polymerase Chain Reaction (PCR) for the Molecular Serotyping of L. monocytogenes

The multiplex PCR was performed by amplifying specific *L. monocytogenes* genes, using primers previously described by [9]. The total reaction volume of 25 µL consisted of 2.5 µL of bacterial DNA, 12.5 µL of 2× QIAGEN Multiplex PCR Master Mix (Qiagen, Hilden, Germany), and 10 µL of a primer mix containing 0.4 µM forward and reverse primers for *lmo1118*, 0.4 µM for *lmo0737*, *orf2819*, and *orf2110*, 0.1 µM for *prs*, and 0.2 µM for *prfA*. The multiplex PCR protocol included an initial pre-denaturation and polymerase activation step at 95 °C for 5 min, followed by 40 amplification cycles consisting of denaturation at 95 °C for 20 s, annealing at 54 °C for 40 s, and elongation at 72 °C for 90 s. A final elongation step was performed at 72 °C for 7 min. For electrophoresis, a 2% agarose gel (Bioline, London, UK) was prepared in TAE buffer (Bioline, UK). Electrophoresis was conducted for 1 h and 30 min at 100 V. A total of 5 µL of DNA ladder (Promega, Southampton, UK) and 10 µL of amplicon were loaded onto the gel, both mixed with 2 µL of 6× dual-action non-toxic fluorescent nucleic acid stain and loading dye (UView 6× Loading Dye, Bio-Rad, Hercules, CA, USA). The gel was visualized alongside a 100 bp DNA ladder for size reference. The expected band sizes for gene identification are detailed in Table 2, while the correlation between the amplified genes and the *L. monocytogenes* serogroups is presented in Table 3.

### 4.6. Data Processing and Visualization

With the aim of analyzing the prevalence of *L. monocytogenes* in the investigated data sets, the Python (version 3.8.8) programming language has been used, specifically employing the Pandas library (version 1.2.4) for data manipulation and Altair (version 5.4.1) for constructing the graphical representations [39,40]. The initial datasets, containing the analysis reports in Excel format, were preprocessed using Pandas for data filtering, categorization, and transformation. Several visualizations in the form of stacked bar charts and radial plots were designed to illustrate the distribution of both total and Listeria-positive samples in regard to several factors such as year, product type, milk heat treatment type, or Listeria serovar type.

## 5. Conclusions

The current study provides the first comprehensive nationwide assessment of *L. monocytogenes* prevalence, serogroup distribution, and antimicrobial resistance in dairy products collected across Romania over a three-year period (2021–2023). Among 10,306 dairy samples analyzed, *L. monocytogenes* was detected in 0.41% of cases, with the highest contamination rates found in ice cream and soft cheeses such as telemea and bellows cheese. The predominant serogroup was IVb—historically associated with clinical cases and major listeriosis outbreaks—followed by IIa and IIb, highlighting both virulence potential and environmental persistence of circulating strains. Antimicrobial susceptibility testing revealed resistance to several key antibiotics, notably oxacillin and trimethoprim-sulfamethoxazole (13.95%), and to first-line agents such as ampicillin and penicillin G (11.62% and 9.30%, respectively). These results raise concerns given the reliance on these antibiotics for effective treatment of invasive listeriosis. The identification of resistance-associated genes such as *tet(M)*, *ampC*, and *dfrD* in other Romanian isolates supports the likelihood of emerging antimicrobial resistance traits in foodborne strains.

Taken together, the results meet the objectives of the study by demonstrating that while overall prevalence remains relatively low, the presence of virulent and potentially resistant *L. monocytogenes* strains in high-risk dairy products poses a significant public health concern. These findings underline the need for continuous surveillance, improved labeling and traceability of thermal processing, and stricter hygienic controls throughout the dairy production chain to minimize the risk of foodborne listeriosis, particularly among vulnerable populations.

To the best of our knowledge, this is the first comprehensive study addressing these issues within the country, supported by extensive data collection and analysis of a wide range of dairy matrices, including fresh and aged cheeses, ice cream, and other dairy-based products.

While this study provides valuable insight into the prevalence, serogroup distribution, and antimicrobial resistance of *L. monocytogenes* in dairy products across Romania, certain limitations must be acknowledged. First, the molecular characterization of isolates was limited to serogroup-level typing; whole-genome sequencing or multilocus sequence typing (MLST) was not performed. As a result, it was not possible to identify the sequence types (STs) of the strains, which would have allowed for more precise comparisons with internationally circulating clones, particularly hypervirulent or outbreak-associated strains reported across Europe. Additionally, the lack of detailed metadata for some samples (e.g., exact production methods, facility hygiene conditions) limited the ability to perform in-depth risk factor analysis.

Future studies should aim to address these gaps by incorporating advanced molecular typing techniques, expanding the range of tested food matrices, and improving documentation of processing conditions to enhance the accuracy of contamination risk assessment.

The study’s results are valuable for comparative analysis with findings from other countries, helping to establish a broader understanding of *L. monocytogenes* contamination trends and resistance profiles.

## Figures and Tables

**Table 1:** Antimicrobial susceptibility of the *L. monocytogenes* isolates.

Antimicrobial Agent	Resistant *L. monocytogenes* Isolates (%)	Intermediate Resistant *L. monocytogenes* Isolates (%)
Ampicillin	5 (11.62%)	1 (2.32%)
Cephalothin	3 (6.97%)	0
Penicillin G	4 (9.30%)	1 (2.32%)
Oxacillin	6 (13.95%)	2 (4.65%)
Methicillin	1 (2.32%)	0
Ciprofloxacin	0	0
Levofloxacin	0	0
Moxifloxacin	0	0
Clindamycin	1 (2.32%)	0
Tetracycline	5 (11.62%)	0
Gentamicin	1 (2.32%)	0
Chloramphenicol	1 (2.32%)	0
Rifampicin	1 (2.32%)	0
Trimethoprim-sulfamethoxazole	6 (13.95%)	1 (2.32%)

**Table 2:** Primers used for the molecular serotyping of *L. monocytogenes*.

Target Gene	Primer Sequence (5′-3′)	Product Size (bp)	Serovar Specificity	Protein Encoded by the Target Gene
*lmo0737*	F:AGGGCTTCAAGGACTTACCC R: ACGATTTCTGCTTGCCATTC	691	*L. monocytogenes* serovars 1/2a, 1/2c, 3a, and 3c	Unknown, no similarity
*lmo1118*	F: AGGGGTCTTAAATCCTGGAA R: CGGCTTGTTCGGCATACTTA	906	*L. monocytogenes* serovars 1/2c and 3c	Unknown, no similarity
*orf2819*	F: AGCAAAATGCCAAAACTCGT R: CATCACTAAAGCCTCCCATTG	471	*L. monocytogenes* serovars 1/2b, 3b, 4b, 4d, and 4e	Putative transcriptional regulator
*orf2110*	F: AGTGGACAATTGATTGGTGAA R: CATCCATCCCTTACTTTGGAC	597	*L. monocytogenes* serovars 1/2b, 3b, 4b, 4d, and 4e	Putative transcriptional regulator
*prs*	F: GCTGAAGAGATTGCGAAAGAAG R: CAAAGAAACCTTGGATTTGCGG	370	*L. monocytogenes* serovars 4b, 4d, and 4e	Putative secreted protein
*prfA*	F: GATACAGAAACATCGGTTGGC R: GTGTAATCTTGATGCCATCAGG	274	*L. monocytogenes* serovars 4b, 4d, and 4e	Putative secreted protein

**Table 3:** Correlation between amplified genes and *L. monocytogenes* serogroups identified by multiplex PCR.

Gene	Serogroup IIa	Serogroup IIb	Serogroup IIc	Serogroup IVb
*lmo0737*	+	−	+	−
*ORF2819*	−	+	−	+
*lmo1118*	−	−	+	−
*ORF2110*	−	−	−	+
*prs*	+	+	+	+
*prfA*	+	+	+	+

## Data Availability

Data is contained within the article.

## Correction Stantement

Due to an error in article production, incorrect references were previously listed in the main text. This information has been updated and this change does not affect the scientific content of the article.

## Abbreviations

The following abbreviations are used in this manuscript:

RTE	Ready-to-eat
EU	European Union
EFSA	European Food Safety Authority
EUCAST	European Committee on Antimicrobial Susceptibility Testing
DNA	Deoxyribonucleic acid
PCR	Polymerase Chain Reaction
